# Seasonal Analysis of Microbial Communities in Precipitation in the Greater Tokyo Area, Japan

**DOI:** 10.3389/fmicb.2017.01506

**Published:** 2017-08-11

**Authors:** Satoshi Hiraoka, Masaya Miyahara, Kazushi Fujii, Asako Machiyama, Wataru Iwasaki

**Affiliations:** ^1^Department of Computational Biology and Medical Sciences, Graduate School of Frontier Sciences, The University of Tokyo Chiba, Japan; ^2^Atmosphere and Ocean Research Institute, The University of Tokyo Chiba, Japan; ^3^Department of Biological Sciences, Graduate School of Science, The University of Tokyo Tokyo, Japan

**Keywords:** microbial ecology, precipitation, long-distance transportation, ice nucleation activity, habitat estimation

## Abstract

The presence of microbes in the atmosphere and their transport over long distances across the Earth's surface was recently shown. Precipitation is likely a major path by which aerial microbes fall to the ground surface, affecting its microbial ecosystems and introducing pathogenic microbes. Understanding microbial communities in precipitation is of multidisciplinary interest from the perspectives of microbial ecology and public health; however, community-wide and seasonal analyses have not been conducted. Here, we carried out 16S rRNA amplicon sequencing of 30 precipitation samples that were aseptically collected over 1 year in the Greater Tokyo Area, Japan. The precipitation microbial communities were dominated by Proteobacteria, Firmicutes, Bacteroidetes, and Actinobacteria and were overall consistent with those previously reported in atmospheric aerosols and cloud water. Seasonal variations in composition were observed; specifically, Proteobacteria abundance significantly decreased from summer to winter. Notably, estimated ordinary habitats of precipitation microbes were dominated by animal-associated, soil-related, and marine-related environments, and reasonably consistent with estimated air mass backward trajectories. To our knowledge, this is the first amplicon-sequencing study investigating precipitation microbial communities involving sampling over the duration of a year.

## Introduction

Microbes are present and move around nearly everywhere in the Earth. Aerial microbes have received considerable attention within this context because the atmosphere not only is an unusual habitat for microbes but also likely represents a path by which microbes move exceptionally long distances (Kellogg and Griffin, 2006; Burrows et al., 2009; Després et al., 2012; Smith, 2013; Fröhlich-Nowoisky et al., 2016). To date, several studies have investigated aerial microbial communities on airborne particles and in clouds using culture-dependent and independent techniques (Bowers et al., 2011a,b, 2013; Vaïtilingom et al., 2012; Zweifel et al., 2012; DeLeon-Rodriguez et al., 2013; Woo et al., 2013; Dong et al., 2016), and revealed that aerial microbes can originate from terrestrial habitats, including plant surfaces (Bowers et al., 2009, 2011a,b). The long-distance transport of aerial microbes has also been reported, for example from Chinese deserts to Japan over the east Eurasian continent and the Sea of Japan (Echigo et al., 2005; Maki et al., 2011). Pathogens in the atmosphere may be transported over long distances, as integrated simulation analyses of climate and disease propagation suggest the involvement of aerial microbes in human diseases (Rodó et al., 2011, 2014). Likewise, the outbreak of several plant infections due to aerial microbes transported beyond borders and seas has been hypothesized (Fitt et al., 1989; Brown and Hovmøller, 2002).

Precipitation, i.e., rainfall and snowfall, would bring aerial microbes in the troposphere to the ground surface. Quantitative polymerase chain reaction (PCR) has detected pathogenic bacterial sequences in precipitation samples (Kaushik et al., 2012), implicating that precipitation may alter microbial ecosystems on the ground (Hervàs et al., 2009; Peter et al., 2014). In the reverse direction, aerial microbes impact the climate by accelerating cloud formation and precipitation, known as “bioprecipitation” (Hamilton and Lenton, 1998; Christner et al., 2008; Konstantinidis, 2014; Morris et al., 2014; Stopelli et al., 2015; Hara et al., 2016). Several microbial species experimentally exhibit ice nucleation activity (INA), which is the ability to accelerate ice nucleation at relatively warm temperatures by producing so-called INA proteins (Hoose and Möhler, 2012). Such INA microbes are broadly distributed among bacteria and fungi and have been isolated from precipitation and cloud water (Mortazavi et al., 2008; Joly et al., 2013). In addition, microbes in clouds may affect the chemical composition of clouds via carbon (Amato et al., 2007; Vaïtilingom et al., 2013) and nitrogen metabolism (Hill et al., 2007). Thus, a basic understanding of microbial communities in precipitation provides important knowledge regarding microbial ecology, public health, and even meteorology. To date, several cloning-based (Ahern et al., 2007; Zweifel et al., 2012; Šantl-Temkiv et al., 2013; Peter et al., 2014) and community-wide but short-term (Cho and Jang, 2014; Kaushik et al., 2014) analyses of microbial communities in precipitation have been carried out. However, community-wide and seasonal analyses have not been conducted.

Here, we conducted 16S ribosomal RNA (rRNA) amplicon-sequencing analysis of 30 precipitation samples that were aseptically collected over 1 year in the Greater Tokyo Area, Japan. Microbial community analysis revealed seasonal variations in their composition. Notably, the estimated original habitats of precipitation microbes showed reasonable consistency with estimated air mass backward trajectories. Our results support a precipitation-mediated microbial cycle model in which soil, oceanic, and animal-associated microbes are spread in the atmosphere, transported for long distances, and deposited via precipitation.

## Materials and methods

### Precipitation sampling

**Figure 1:**
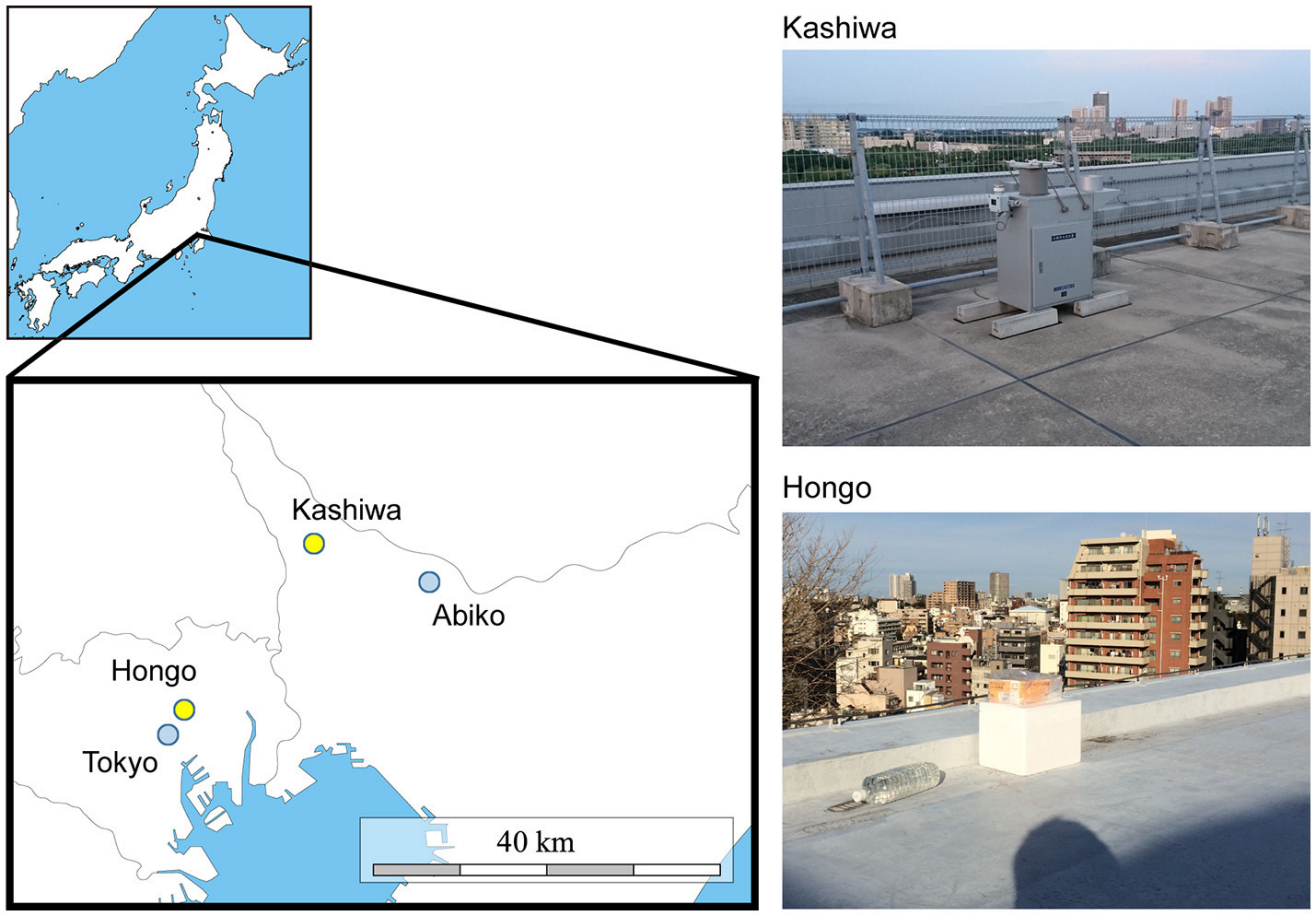
A map of the sampling sites (Kashiwa and Hongo, yellow) and meteorological observatories (Abiko and Tokyo, blue) (left panel), with photos of the sampling sites (right panel). At the Kashiwa site, a US-330 automatic precipitation sampler (Ogasawara Keiki, Tokyo, Japan) was installed. At the Hongo site, precipitation samples were manually collected.

Precipitation samples were collected at two sites in the Greater Tokyo Area, Japan: Kashiwa (35°54′00″N, 139°55′59″E, 50 m above sea level) and Hongo (35°42′55”N, 139°45'56″E, 30 m above sea level) (Figure 1). The Kashiwa site was on the roof of a seven-story building on the Kashiwa campus, the University of Tokyo, Chiba, Japan, which is surrounded by residences, farms, and woods in a suburb of Tokyo. The Hongo site was on the roof of a five-story building on the Hongo campus, the University of Tokyo, Tokyo, Japan, which is located in downtown Tokyo. The sites are 25.5 km apart and neither geologically nor meteorologically separated in the Kanto plain. The upper areas of both sites are wide open and lack any obstructing buildings or structures that would contaminate the precipitation samples. At the Kashiwa site, precipitation was aseptically collected using a US-330 automatic precipitation sampler (Ogasawara Keiki, Tokyo, Japan) following the method of Kaushik et al. (2012). This device consists of a sterile and disposable bottle inside a 4°C refrigerator and automatically collects precipitation by opening the lid only when a sensor detects precipitation. At the Hongo site, precipitation samples were manually collected into a sterile and disposable bottle on ice and immediately stored in a 4°C refrigerator. At both sites, every part of the collection equipment that potentially directly contacted precipitation samples (e.g., disposable collection bottles and channel tubes) was sterilized by gamma rays in advance of each sample collection. The precipitation samples were pre-filtered through 5-μm membrane filters, and microbial cells were collected using 0.22-μm Sterivex filters (Millipore, USA). The Sterivex filters were promptly moved to a −20°C freezer and stored until DNA extraction. Precipitation sampling required no special permission. To prepare negative control samples, we poured 1 L of Milli-Q purified water into the collection equipment and carried out filtration in the same manner.

We collected 25 and 5 precipitation samples containing sufficient amounts of microbial DNA at the Kashiwa and Hongo sites, respectively. The sampling dates spanned more than 1 year from May 2014 to October 2015, encompassing the rainy and typhoon seasons in Japan (Table 1; the six digits, letter, and suffix number for each sample name represent the sampling date (YYMMDD), the site (K for Kashiwa and H for Hongo), and the volume (if multiple samples were collected during the same precipitation event). A precipitation event was defined if there was no precipitation 6 h before and after the event. The volumes of collected and filtered precipitation ranged from 50 to 1,000 mL. For correlation analysis with meteorological data, we excluded the data obtained from samples 140630K_50, 140630K _100, 140810K_50, and 140810K_100, which were retrieved as replicate samples with different volumes. Eight negative control samples were also collected at different dates at the Kashiwa and Hongo sites.

**Table 1:** Sequencing statistics and meteorological characteristics of each precipitation sample.

**Sample**	**Sampling time (YYMMDD)^a^**	**Note**	**Amount of precipitation [mm]**	**Temperature [°C]**	**Atmospheric pressure**	**Wind speed [m/s]**	**Filtration volume [mL]**	**Raw reads**	**Effective reads**	**OTUs**	**Shannon's diversity index**
140521K	140521(01:00)–140521(18:00)	–	36	16.56	994.31	3.16	50	8,340	246	44	2.62
140630K_50	140628(01:00)–140630(05:00)	Rainy season	22	21.98	999.23	1.74	50	8,622	1,092	27	1.63
140630K_100	140628(01:00)–140630(05:00)	Rainy season	22	21.98	999.23	1.74	100	7,444	1,287	39	1.26
140630K_200	140628(01:00)–140630(05:00)	Rainy season	22	21.98	999.23	1.74	200	7,462	1,118	44	1.48
140810K_50	140810(00:00)–141810(23:00)	Typhoon	31.5	25.46	998.46	3.89	50	7,621	275	76	3.70
140810K_100	140810(00:00)–141810(23:00)	Typhoon	31.5	25.46	998.46	3.89	100	8,441	108	53	4.41
140810K_200	140810(00:00)–141810(23:00)	Typhoon	31.5	25.46	998.46	3.89	200	6,664	371	129	3.82
140926K	140925(02:00)–140926(04:00)	–	6.5	21.48	1,001.08	2.14	200	1,941	18	15	2.66
141014K	141013(13:00)–141014(07:00)	Typhoon	32.5	19.58	992.99	4.47	200	1,641	317	157	4.76
141023K	141021(05:00)–141023(18:00)	–	31.5	14.89	1,010.85	2.03	200	1,354	120	54	3.66
150107K	150106(16:00)–150106(18:00)	–	4	12.35	992.30	5.10	200	3,410	37	22	2.93
150116K	150115(11:00)–150116(00:00)	–	40.5	4.97	1,005.15	3.05	1,000	2,494	72	55	3.86
150202K	150130(05:00)–150130(19:00)	Snow	12.5	1.11	1,015.18	2.17	400	9,705	1,256	194	4.69
150409K	150407(03:00)–150408(17:00)	–	20.5	6.39	1,017.32	2.13	200	8,337	473	111	4.15
150412K	150410(17:00)–150411(14:00)	–	16	8.99	1,018.06	1.60	200	6,805	771	125	4.16
150414K	150413(11:00)–150414(17:00)	–	36.5	10.13	1,013.38	1.91	200	5,818	655	116	3.98
150513K	150512(21:00)–150513(01:00)	Typhoon	23	20.00	997.43	5.60	200	2,223	47	25	3.92
150604K	150603(08:00)–150603(13:00)	Rainy season	13	20.46	998.26	1.44	200	4,663	8	7	2.88
150628K	150626(19:00)–150627(12:00)	Rainy season	13.5	20.92	997.42	1.24	150	9,634	284	46	3.41
150711K	150708(15:00)–150709(20:00)	Rainy season	17.5	19.27	1,013.22	1.48	200	9,557	31	20	1.91
150718K	150716(04:00)–150717(13:00)	Typhoon	16.5	25.99	1,004.62	3.26	200	5,189	5	4	4.03
150816K	150814(05:00)–150814(22:00)	–	43	25.11	1,000.38	1.91	200	6,864	269	68	3.05
150827K	150826(00:00)–150826(17:00)	Typhoon	27	20.10	1,005.27	1.92	200	7,993	1,041	226	3.39
150926K	150924(19:00)–150926(06:00)	–	21	17.63	1,005.28	1.90	200	2,988	6	4	2.75
151014K	151011(01:00)–151011(11:00)	–	8	16.94	1,008.92	1.00	200	6,357	129	30	1.33
150414H	150413(07:00)–150414(12:00)	–	39.5	10.03	1,014.99	3.15	200	7,215	882	125	3.76
150513H	150512(20:00)–150513(06:00)	Typhoon	58.5	20.25	997.43	7.23	200	3,198	59	38	4.83
150627H	150626(15:00)–150627(10:00)	Rainy season	16	21.33	998.27	2.22	150	6,450	667	108	1.24
150710H	150708(10:00)–150710(00:00)	Rainy season	22	20.20	1,013.22	2.33	200	9,280	159	48	3.00
151014H	151011(02:00)–151011(10:00)	–	15	18.01	1,008.80	1.90	200	6,342	286	67	3.70

a *The six digits, letter, and suffix number in each sample name represent the sampling date (YYMMDD), the sampling site (K for Kashiwa and H for Hongo), and the filtered sample volume if prepared as a technical replicate with multiple volume sizes (50, 100, and 200 mL)*.

### DNA extraction and PCR amplification

Microbial DNA on the Sterivex filters was retrieved using a ChargeSwitch Forensic DNA Purification Kit (Invitrogen) according to the supplier's protocol with one exception: the filters were directly suspended in the extraction solution from the kit during the cell lysis process. The V5-V6 region of the prokaryotic 16S rRNA gene was amplified using a standard PCR protocol with TaKaRa Ex Taq (TaKaRa) and the following high-performance liquid chromatography-purified primers: 784F (5′- RGGATTAGATACCC -3′) and 1064R (5′- CGACRRCCATGCANCACCT -3′) (Wang and Qian, 2009; Claesson et al., 2010). Amplified DNA was concatenated to multiplex identifier tags that were unique to each sample, and a mixture of 10 samples on average was sequenced in one run on a 454 GS Junior System (Roche) after size selection (350 ± 50 bp). Pre-packaged sterile water for injection (*in lieu* of water from a laboratory water purification system) was used throughout the DNA extraction, PCR amplification, and DNA library preparation steps to avoid water-mediated contamination.

### Bioinformatic analysis

For raw sequence data from both precipitation and negative control samples, sequence regions at both ends that contained low-quality bases (quality score < 20) were trimmed using DynamicTrim (Cox et al., 2010), chimeric sequences were filtered out using UCHIME with default settings (Edgar et al., 2011), and sequences whose lengths were shorter than 150 bp were discarded. All remaining high-quality sequences were clustered with a 97% identity threshold using CD-HIT (Fu et al., 2012). After discarding clusters that contained negative control sequences (Cho and Jang, 2014), each cluster was designated as an operational taxonomic unit (OTU). For hierarchical cluster analysis of the precipitation samples, the Ward method was used based on Bray-Curtis dissimilarities between their OTU compositions. Non-metric multidimensional scaling (NMDS) analysis was conducted using Bray-Curtis dissimilarities. The taxonomic assignment of each OTU was performed by conducting a BLASTN search (Camacho et al., 2009) against the SILVA database (Quast et al., 2013) and retrieving the top hit sequence that showed *e*-values ≤ 1E-15. To estimate ordinary habitats for each 16S rRNA sequence, a BLASTN search was performed against MetaMetaDB (Yang and Iwasaki, 2014), and the top hit sequence with an *e*-value ≤ 1E-10 and an identity ≥ 90% was retrieved. Microbial habitability index (MHI) scores were calculated as previously described (Yang and Iwasaki, 2014).

Amplicon-sequencing data of aerosol and cloud water samples were downloaded from NCBI SRA database (the accession numbers are shown in Supplementary Table S1). Their ordinary habitat analyses were conducted as described above after quality filtering.

### Meteorological data analysis

The data on the amount of precipitation, temperature, wind speed, and atmospheric pressure were retrieved from the website of the Japan Meteorological Agency (http://www.jma.go.jp/jma/menu/menureport.html), Ministry of Land, Infrastructure, and Transport of Japan. Precipitation, wind speed, and temperature data from the Abiko (35°51′48″N, 140°06′36″E; 16.4 km from Kashiwa) and Tokyo (35°41′30″N, 139°45′00″E; 2.9 km from Hongo) observatories were used for the analyses of the Kashiwa and Hongo sites, respectively (Figure 1). Atmospheric pressure data from the Tokyo observatory were used (this observatory is the closest to both sites that records atmospheric pressure data). The wind speed, temperature, and atmospheric pressure data were averaged over the period of each precipitation event. To analyze long-range transport paths of air masses that caused precipitation by providing water vapor, we estimated backward trajectories of an air mass at 2,000 m altitude for 240 h prior to all precipitation events for each sampling site. The trajectories were calculated based on the hybrid single-particle Lagrangian integrated trajectory (HYSPLIT) model (http://ready.arl.noaa.gov/HYSPLIT.php) provided by the Global Data Assimilation System of National Oceanic and Atmospheric Administration, USA (Stein et al., 2015). The HYSPLIT model uses gridded meteorological data and considers advection and diffusion of air parcels in calculation of their trajectories. This model has been used in a variety of atmospheric simulations focusing on the atmospheric transport, dispersion, and deposition of pollutants and hazardous materials (Stein et al., 2015), while it has also been adopted for estimation of sources of airborne microbes (e.g., Smith et al., 2013; Cho and Jang, 2014; Kobayashi et al., 2015; Xia et al., 2015; Xu et al., 2017).

### Data deposition

The amplicon sequence data were deposited in the DDBJ/ENA/GenBank database under BioSample IDs SAMD00059586-SAMD00059614 and SAMD00060461-SAMD00060468. All data were registered under BioProject ID PRJDB5087.

## Results and discussion

### Amplicon sequencing of precipitation samples

A total of 64,100 high-quality sequences 231 ± 45 bp in length were generated from 30 precipitation and eight negative control samples. The precipitation samples included typhoon rain, rainy season rain, and snow. After removing sequences exhibiting >97% similarity to the negative control samples, 12,089 “effective” sequences comprising 1,297 OTUs remained. To make our analyses based on reads that were not likely from contamination as much as possible, we took a conservative and strict filtering approach, whose extent of read number reduction was similar to that in a previous study (Cho and Jang, 2014). The number of OTUs per sample ranged from 4 to 226 (Table 1). Based on rarefaction curves, the obtained OTUs represented their microbial communities well for some samples, although several samples required additional sequences (Supplementary Figure S1).

**Figure 2:**
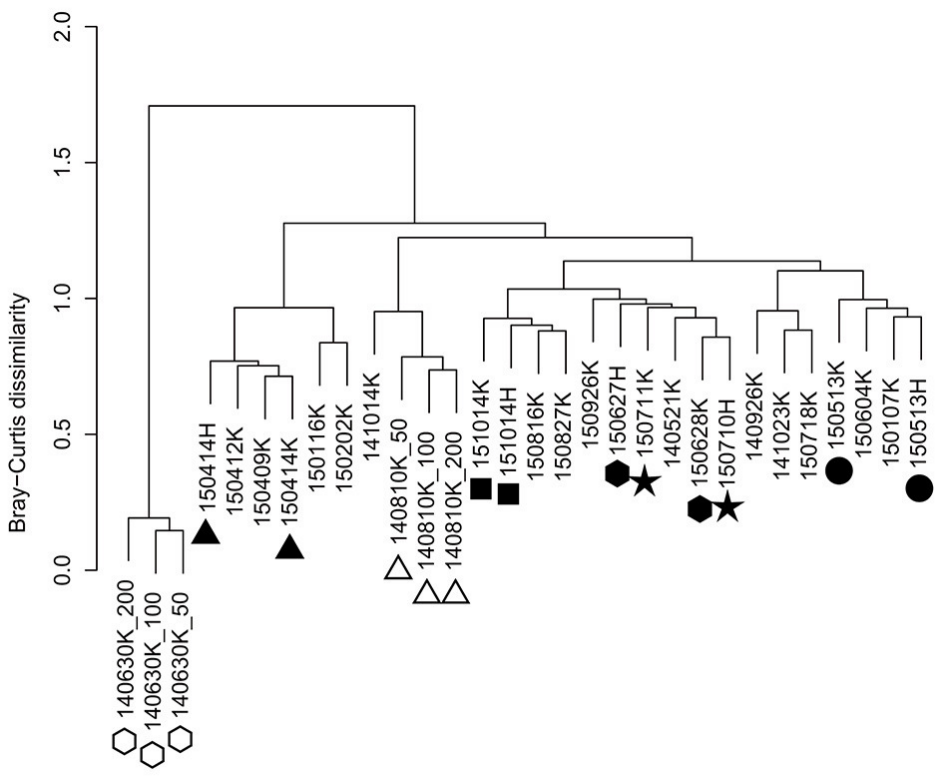
Hierarchical clustering of precipitation samples based on OTU composition. The distance matrix was calculated based on the Bray-Curtis dissimilarity, and clusters were calculated using Ward's method. Open symbols indicate samples that were collected during the same precipitation event with different volumes. Closed symbols indicate samples that were collected on the same day at different sites (Kashiwa and Hongo).

Hierarchical cluster analysis of OTU composition in the precipitation samples indicated samples collected during the same precipitation event with different volumes (50, 100, and 200 mL) that were highly similar to each other (Figure 2, open symbols), suggesting that differences in volume have little effect on analysis in the 50–200 mL range. Moreover, microbial communities in samples that were collected on the same day at different sampling sites (Kashiwa and Hongo) were closely positioned in the dendrogram (Figure 2, closed symbols), indicating that the observed OTU compositions reflect the microbial populations in precipitation rather than those in the atmosphere near the ground surface or equipment- or reagent-mediated contamination at each site. NMDS analysis did not show any clear trend, although samples of close dates tended to be clustered together (Supplementary Figure S2).

### Taxonomic composition of precipitation microbial communities

**Figure 3:**
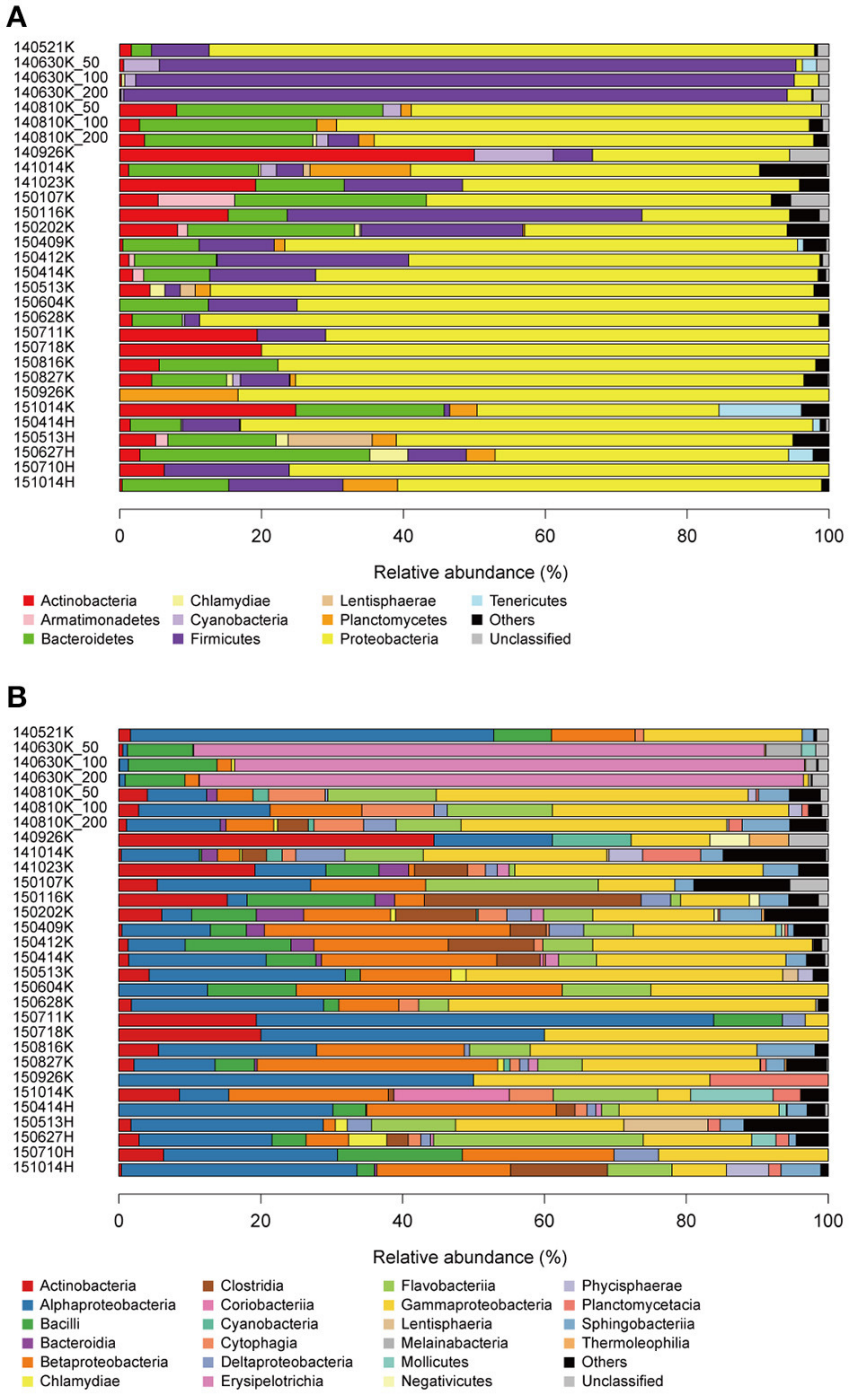
Relative abundances of sequences at the phylum **(A)** and class **(B)** levels. Groups demonstrating <5% abundance were summarized as “Others.”

Among the 12,089 effective sequences, 11,994 (99.2%) were taxonomically assigned at the phylum level. Almost all sequences were assigned to 24 phyla in the domain Bacteria with the exception of 4 (0.03%) and 219 (1.7%) sequences assigned to Archaea and mitochondria, respectively. This strong bias toward bacterial sequences may reflect the actual composition but may also be attributable to amplification bias introduced by primer specificity. The top three and six most abundant bacterial phyla accounted for >80 and >95%, respectively, of the sequence pool of all precipitation samples (Figure 3A). Proteobacteria was the most abundant phylum (23–88%) across all precipitation samples with the exception of the 140630, 140926, and 150116K samples (Firmicutes (89–94%), Actinobacteria (50%), and Firmicutes (49%) were the most abundant phyla, respectively). A particularly exceptional microbial community dominated by Firmicutes was observed in the 140630K sample. Firmicutes, Bacteroidetes, and Actinobacteria were the other dominant phyla in the total sequence pool. In principle, these results were consistent with those of a previous study in which Proteobacteria, Firmicutes, and Bacteroidetes were the dominant phyla in precipitation samples captured in Seoul, Korea (Cho and Jang, 2014), whereas comparatively greater numbers of sequences were assigned to Actinobacteria, Planctomycetes, and Cyanobacteria in this study. At the class level, the abundant groups were Gammaproteobacteria, Betaproteobacteria, and Alphaproteobacteria, followed by Bacilli, Flavobacteriia, Clostridia, Actinobacteria, and Sphingobacteriia (Figure 3B). Notably, the enrichment of these phyla and classes was also reported in previous studies investigating aerosolized (Bowers et al., 2009, 2011a; DeLeon-Rodriguez et al., 2013) and cloud water microbial communities (Kourtev et al., 2011; DeLeon-Rodriguez et al., 2013).

Several OTUs were assigned to genera that potentially contain INA bacteria, i.e., *Acinetobacter, Bacillus, Erwinia, Flavobacterium, Luteimonas, Microbacterium, Pseudomonas, Psychrobacter, Sphingomonas*, and *Stenotrophomonas* (Després et al., 2012). We also detected several genera containing known pathogens, including typical human pathogens such as *Legionella, Streptococcus, Arcobacter, Rickettsia*, and *Clostridium*, and plant pathogens such as *Erwinia*, although their abundance was low. We did not detect season-specific microbial groups in the typhoon rain, rainy season, and snow samples with statistical significance, probably partly due to small sample sizes.

### Seasonal and meteorological correlations

**Figure 4:**
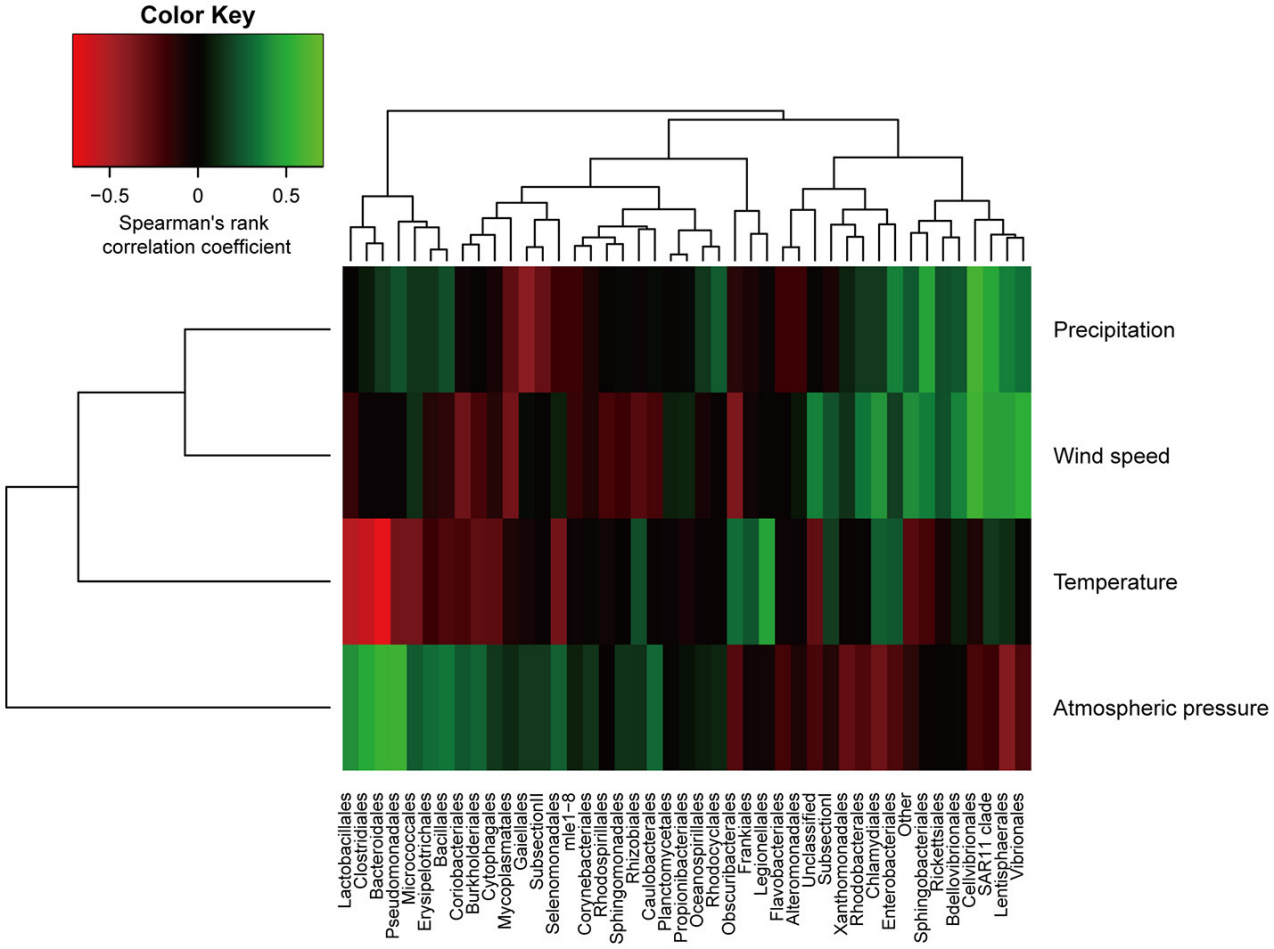
Correlation analysis between relative abundances of sequences at the order level and meteorological data. The color scheme represents Spearman's rank correlation coefficient.

Taxonomic distribution exhibited seasonal variability (Figure 3). Notably, the abundance of Proteobacteria decreased from summer to winter (*p* < 0.01, Mann-Whitney *U*-test), and a similar trend has consistently been observed in aerosolized microbial communities (Bowers et al., 2011b). To more closely investigate the factors underlying changes in the precipitation microbial communities, we performed a correlation analysis between meteorological characteristics and microbial composition (Figure 4). The relative abundance of the order Bacteroidales negatively correlated with temperature (Spearman correlation ρ = −0.70, *p* < 0.01 after the Bonferroni correction). Although other correlations were not statistically significant after multiple testing correction, the amount of precipitation, wind speed, and atmospheric pressure showed tendencies of positive correlations with the abundance of the orders Cellvibrionales (ρ = 0.59), Cellvibrionales (ρ = 0.58), and Pseudomonadales (ρ = 0.57), respectively. Notably, the abundance of the order Legionellales, which contains several known pathogens, showed a tendency of a positive correlation with temperature (ρ = 0.47), where aerosolized water is known to facilitate the dispersion of *Legionella* (Nguyen et al., 2006) and a warm and wet climate is associated with the incidence of Legionnaires' disease (Fisman et al., 2005; Fisman, 2007). Although cell numbers were not measured except for one sample in this study, we note that seasonal variability in cell numbers would also be important, especially because that of atmospheric samples was reported (Kaushik et al., 2014; Dong et al., 2016). Similarly, analyses with particulate matter density and O_3_ and NO_3_ concentrations are also envisioned, because they would substantially affect aerial microbes (Kaushik et al., 2012; DeLeon-Rodriguez et al., 2013; Wei et al., 2016; Xu et al., 2017).

### Relationship between ordinary habitats of precipitation microbes and air mass backward trajectories

**Figure 5:**
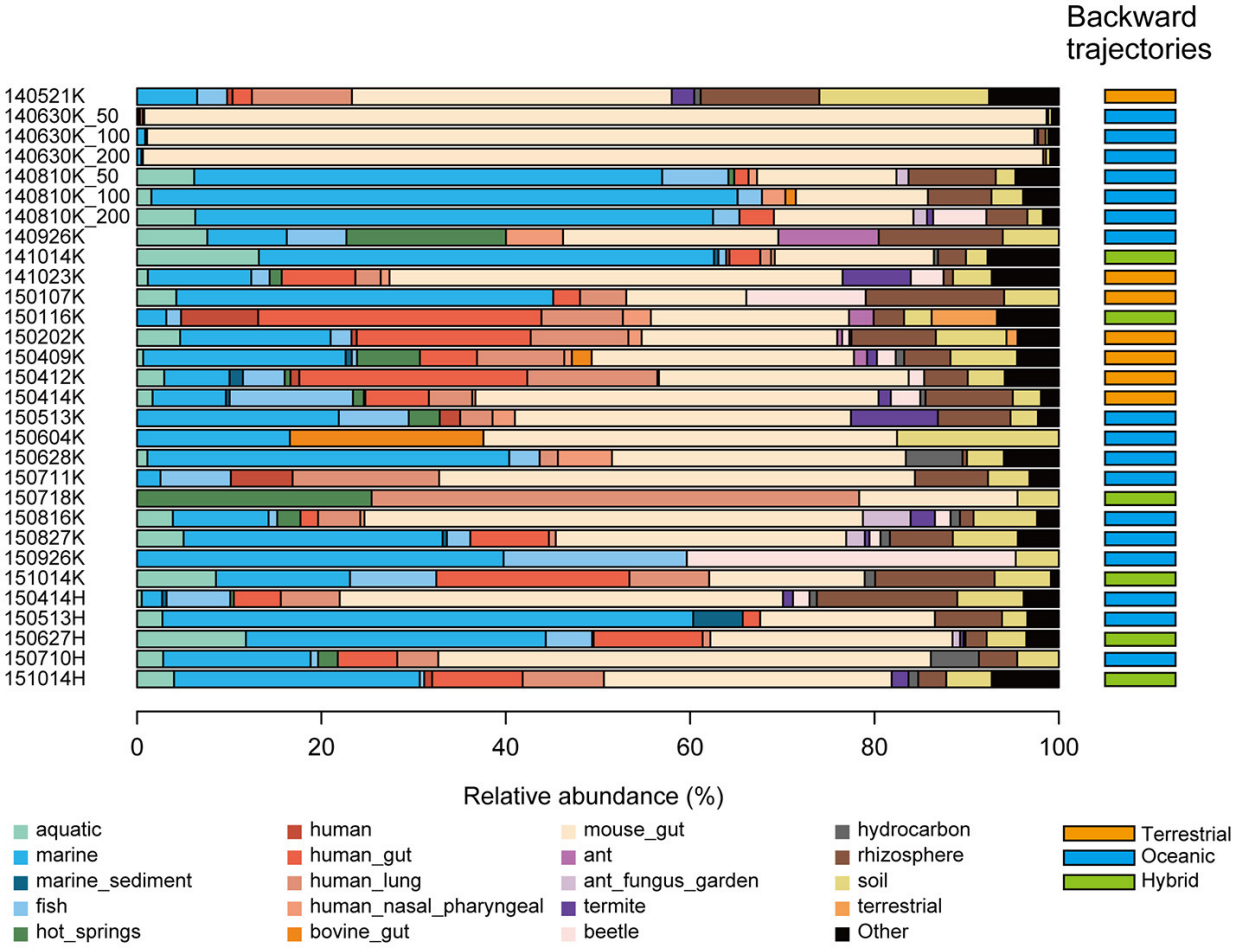
Estimated ordinary habitats of precipitation microbes. Because the ordinary habitat for an individual 16S rRNA sequence cannot be conclusively determined, the microbial habitability index (MHI) was calculated to estimate the probability of an ordinary habitat (Yang and Iwasaki, 2014). Estimated ordinary habitats demonstrating <5% abundance were summarized as “Others.” The estimated route of the air mass before each precipitation event is indicated in the right column. The terrestrial, oceanic, and hybrid routes are colored in orange, blue, and green, respectively. The estimated air mass backward trajectory maps are provided in Supplementary Figure S3.

To estimate the environments from which microbes in precipitation originated, we performed a microbial habitat index analysis using MetaMetaDB (Yang and Iwasaki, 2014), which is a database to estimate the ordinary habitats of microbes based on similarity searches for 16S rRNA gene sequences against amplicon-sequencing and shotgun metagenomic data in public databases. In most samples, animal-associated environments, such as gut microbiota, were estimated to be the most dominant ordinary habitats (52% on average) (Figure 5, Supplementary Figure S4), which is consistent with a previous study in which animal feces were the dominant source of airborne bacteria (Bowers et al., 2011b). Notably, marine-related environments, such as marine and marine sediment, were estimated to be relatively major ordinary habitats for several samples (e.g., 65.1 and 63.1% in the 140810 and 141014K samples, respectively). Soil-related environments, such as soil and rhizosphere, were also estimated to be major ordinary habitats (11.0% on average). For comparison, we also conducted ordinary habitat analyses using amplicon-sequencing data from aerosol (Xia et al., 2015) and cloud water (DeLeon-Rodriguez et al., 2013) samples. The soil-related and animal-associated environments were generally major ordinary habitats as consistent to the present results, whereas marine-related environments were not major possibly because the origins of the microbes or the sampling methods were different from those in our study (Supplementary Figure S5).

The estimated backward trajectories of air masses that led to the precipitation events at the Kashiwa and Hongo sites were classified as terrestrial, oceanic, and hybrid routes. The terrestrial route typically originated from the middle of the Eurasian continent and passed through the East China Sea, the Yellow Sea, and the Sea of Japan; the oceanic route typically originated from the Pacific Ocean and passed through the East China Sea or the Sea of Okhotsk; and the hybrid route comprised both the terrestrial and oceanic areas. Consistent with the typical pattern of the seasonal winds in Asia, the terrestrial and oceanic routes dominated in winter and summer, respectively (Figure 5, Supplementary Figure S3). The estimated ordinary habitats of the precipitation microbes showed agreement with the estimated air mass backward trajectories. For example, Planctomycetes, which contains several aquatic microbes (Fuerst, 1995), was frequently found when the backward trajectories followed oceanic routes (Figures 3A, 5). PERMANOVA analysis showed a significant relationship between the routes and the estimated composition of ordinary microbial habitats (*p* < 0.05). Notably, the ratios of marine-related environments dominated when the air masses originated from the oceanic route, and animal-related environments dominated when they originated from the terrestrial route. Shannon's diversity indices of microbes became larger when the air masses originated from the terrestrial route (Shannon's diversity indices were 3.74 ± 0.68, 3.05 ± 1.00, and 3.15 ± 1.36 for the terrestrial, oceanic, and hybrid routes, respectively. The index of each sample is shown in Table 1); however, it should be noted that some samples required additional sequences to reach plateaus of rarefaction curves as mentioned already.

Soil, oceanic, and animal-associated microbes are spread in the atmosphere and transported for long distances (Morris et al., 2014; Smets et al., 2016), and precipitation may facilitate this microbial cycle. Sea-living microbes are emitted into the atmosphere via the bursting of bubbles on waves (Fahlgren et al., 2015), whereas soil-living and animal-associated microbes are transported on soil dust (Echigo et al., 2005; Prospero et al., 2005; Maki et al., 2011; Yamaguchi et al., 2014). In high-altitude atmospheric environments, microbes may be under substantial selection pressure due to harsh chemical, physical, and nutrient conditions (Delort et al., 2010; Morris et al., 2013; Smith, 2013). INA microbes play roles in cloud formation (Morris et al., 2013) and may facilitate the return of aerial microbes to diverse environments. The dispersal of pathogenic microbes causes disease epidemics that threaten public health and agricultural plant and animal health (Brown and Hovmøller, 2002; Rodó et al., 2011, 2014; Cao et al., 2014). Continuous long-term monitoring and large-scale analysis of precipitation microbes is thus envisioned to reveal the full impact of atmospheric microbial transport on microbial ecology, microbial evolution, public health, and climate.

## Conclusion

Microbes are present nearly everywhere in the Earth, even in precipitation from the sky. Precipitation is supposed to make microbes in the atmosphere finally fall down to the ground surface. In this study, we thoroughly observed microbial communities in precipitation samples that were collected over 1 year in the Grate Tokyo area, Japan. To our knowledge, this is the first amplicon-sequencing study investigating precipitation microbial communities involving sampling over the duration of a year. Most importantly, our results suggest seasonal variations in the microbial communities in precipitation, and their community structures were significantly associated with the estimated air mass trajectories. These results highlight importance of precipitation in long-range microbial immigration via the atmosphere, which may answer how tiny microbes can dynamically travel around the globe.

## Author contributions

SH designed and performed the bioinformatics analyses and wrote the manuscript. MM designed the experiments and performed the sample collection, DNA extraction, DNA sequencing, and bioinformatics analyses. KF and AM designed the experiments and performed the sample collection, cell counting, DNA extraction, and DNA sequencing. WI conceived of and designed the study, wrote the manuscript, and supervised the project. All authors read and approved the final manuscript.

### Conflict of interest statement

The authors declare that the research was conducted in the absence of any commercial or financial relationships that could be construed as a potential conflict of interest.

## Supplementary material

Supplementary Figure S1 Rarefaction curves for each precipitation sample.

Supplementary Figure S2 Nonmetric multidimensional scaling plot for OTU compositions. The distance matrix was calculated based on the Bray-Curtis dissimilarity. The stress value of the final configuration was 20.46%.

Supplementary Figure S3 The estimated air mass backward trajectories 240 h prior to precipitation events.

Supplementary Figure S4 Estimated ordinary habitats of precipitation microbes for three ecosystem groups. The abundance values in each ecosystem group are summation for habitats described below. Marine-related: “aquatic”, “marine”, “marine sediment”, “fish”, and “hot spring”; Animal-associated: “human”, “human gut”, “human lung”, “human nasal pharyngeal”, “bovine gut”, and “mouse gut”; and Soil-related: “hydrocarbon”, “rhizosphere”, “soil”, and “terrestrial.” The estimated route of the air mass before each precipitation event is indicated in the right column.

Supplementary Figure S5 Estimated ordinary habitats of microbes in aerosol and cloud water samples. Estimated ordinary habitats demonstrating <5% abundance were summarized as “Others.”

Supplementary Table S1 Amplicon-sequencing data of aerosol and cloud water samples.
