# Supplementary figures and images for: Seasonal Analysis of Microbial Communities in Precipitation in the Greater Tokyo Area, Japan
Source: Front Microbiol. 2017 Aug 11;8:1506. doi: 10.3389/fmicb.2017.01506 (PMC5554504; doi:10.3389/fmicb.2017.01506)

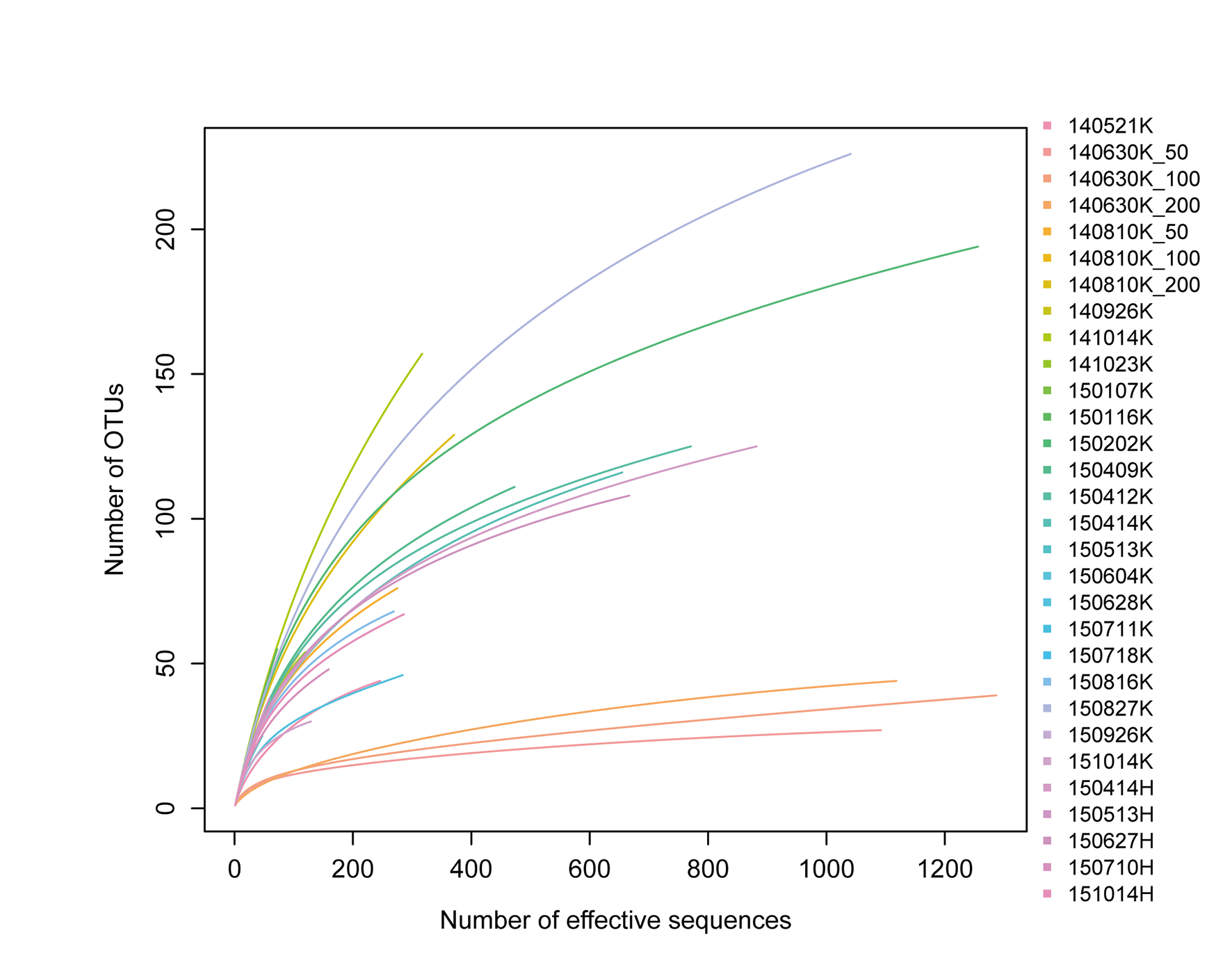

Supplement: Supplementary Figure S1 — Rarefaction curves for each precipitation sample. [file Image1.TIF]

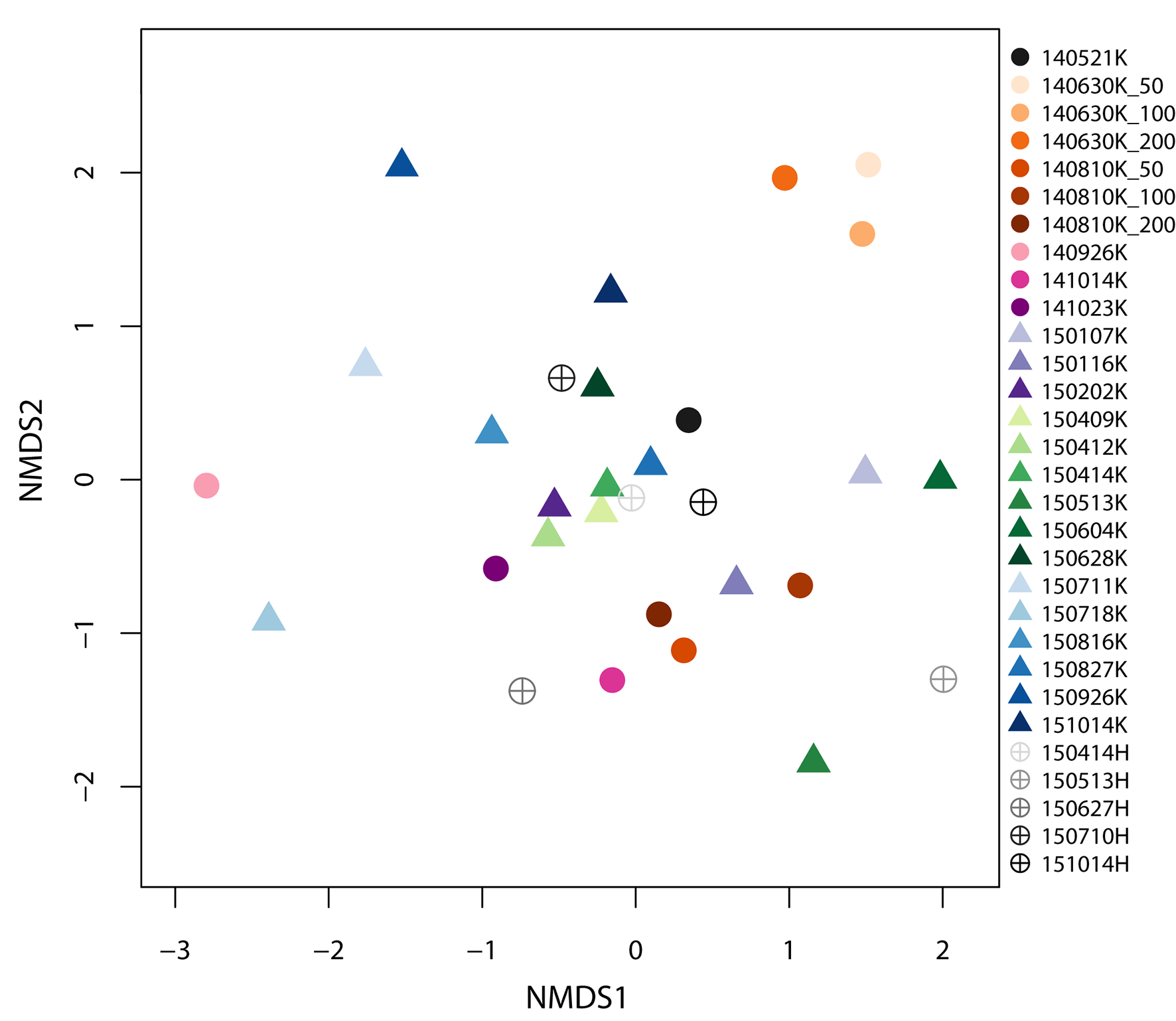

Supplement: Supplementary Figure S2 — Nonmetric multidimensional scaling plot for OTU compositions. The distance matrix was calculated based on the Bray-Curtis dissimilarity. The stress value of the final configuration was 20.46%. [file Image2.TIF]

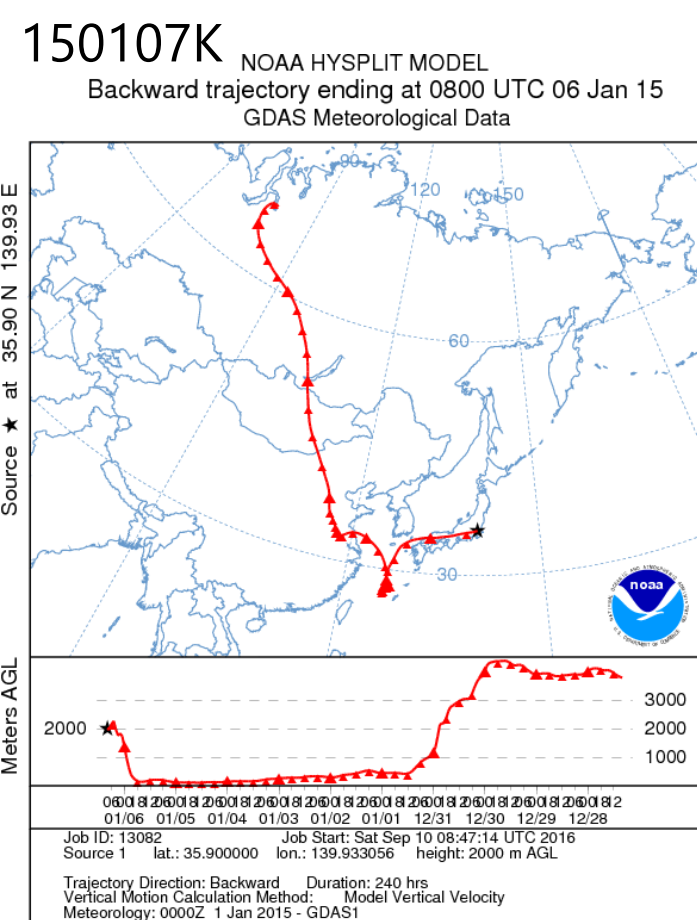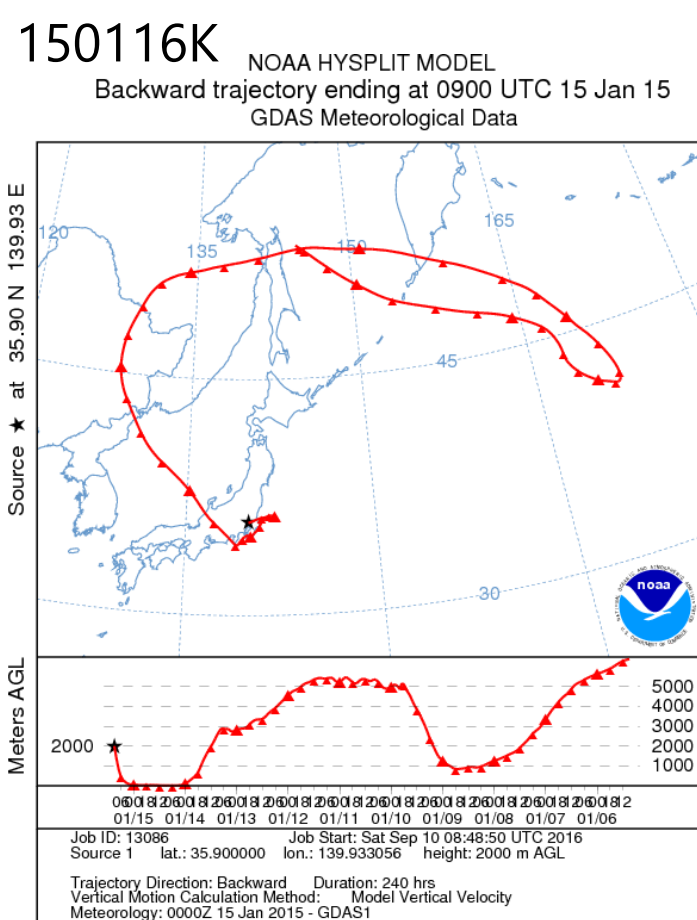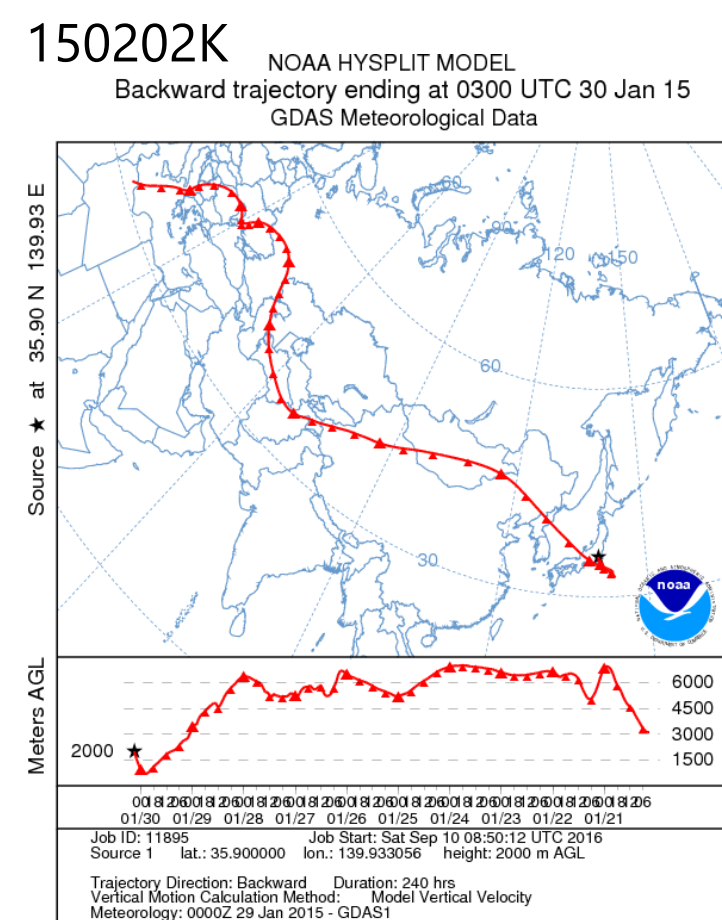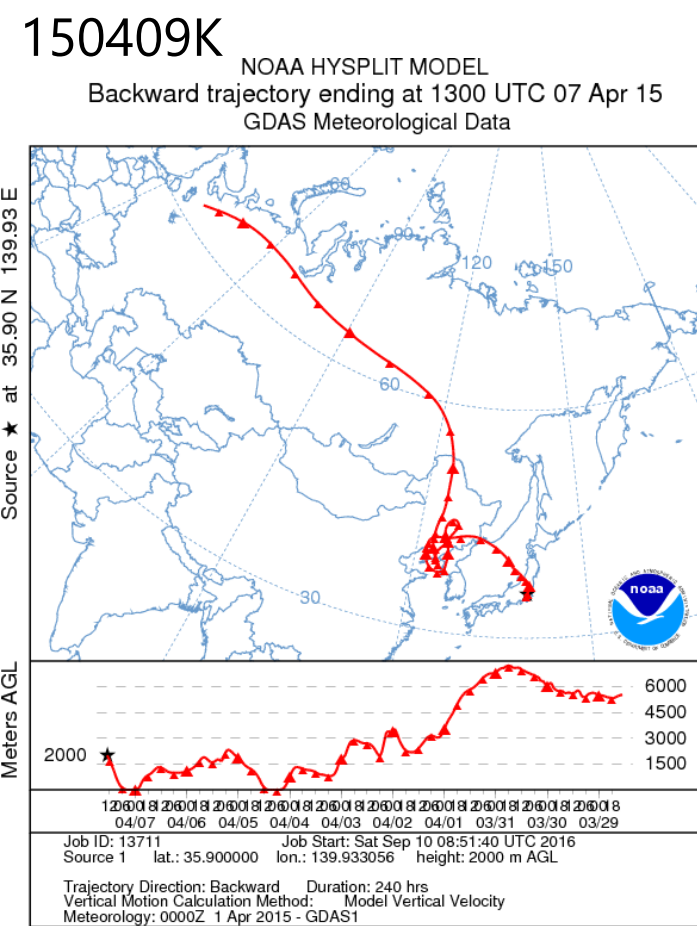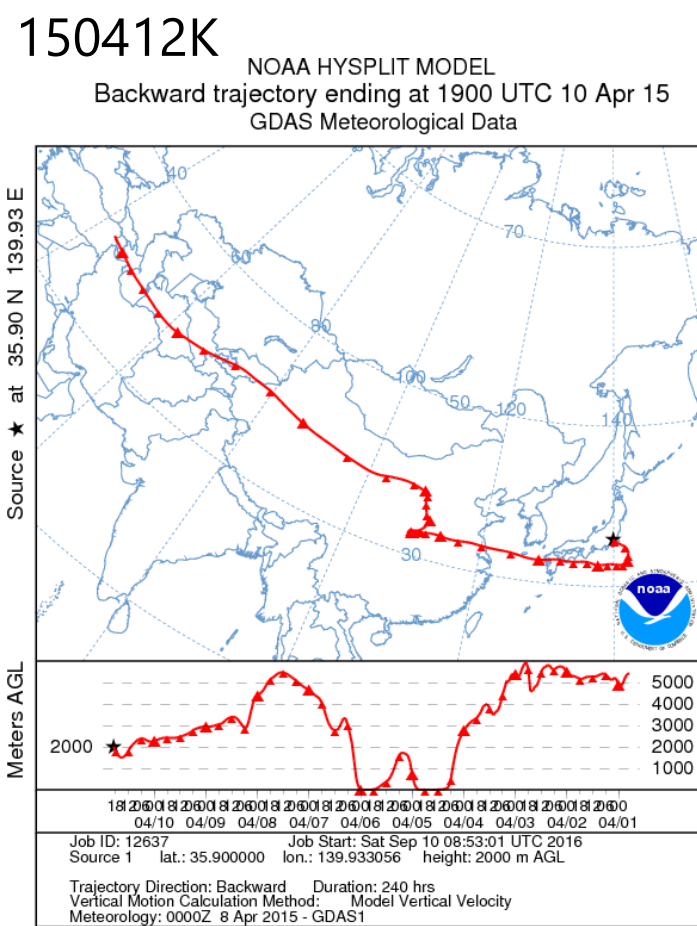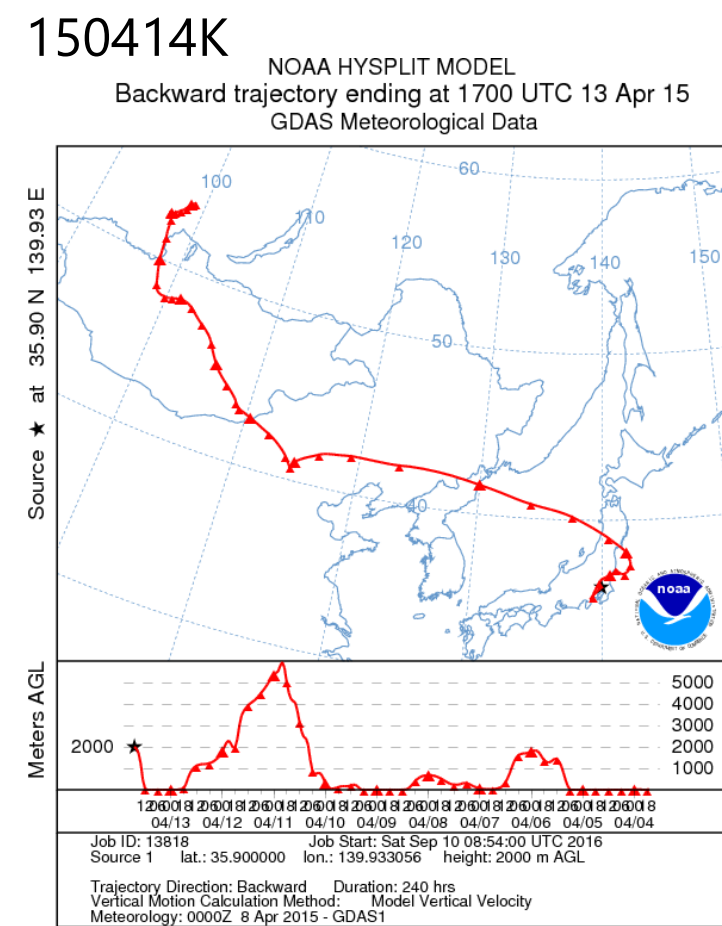

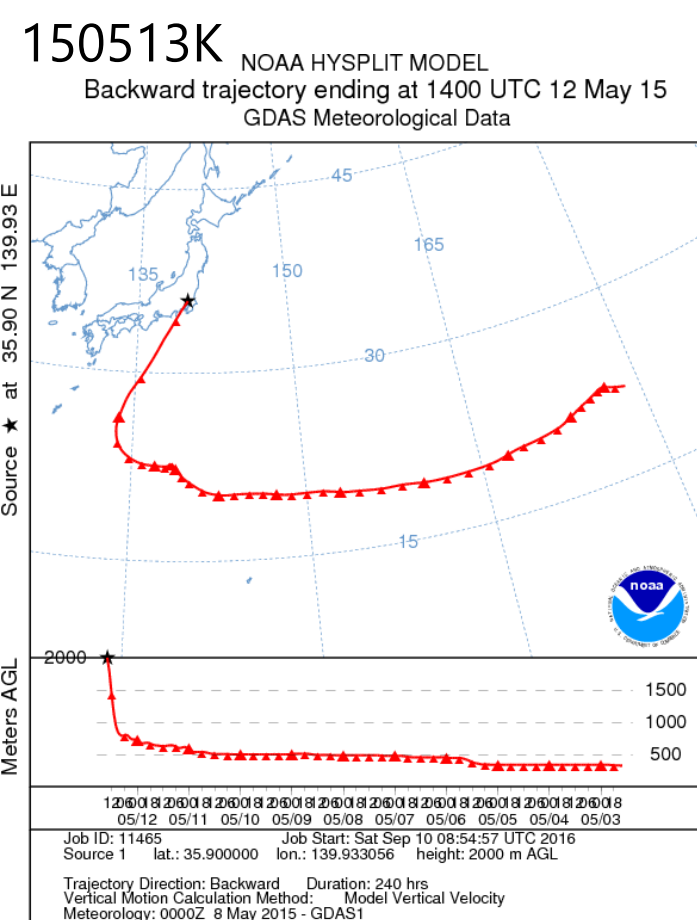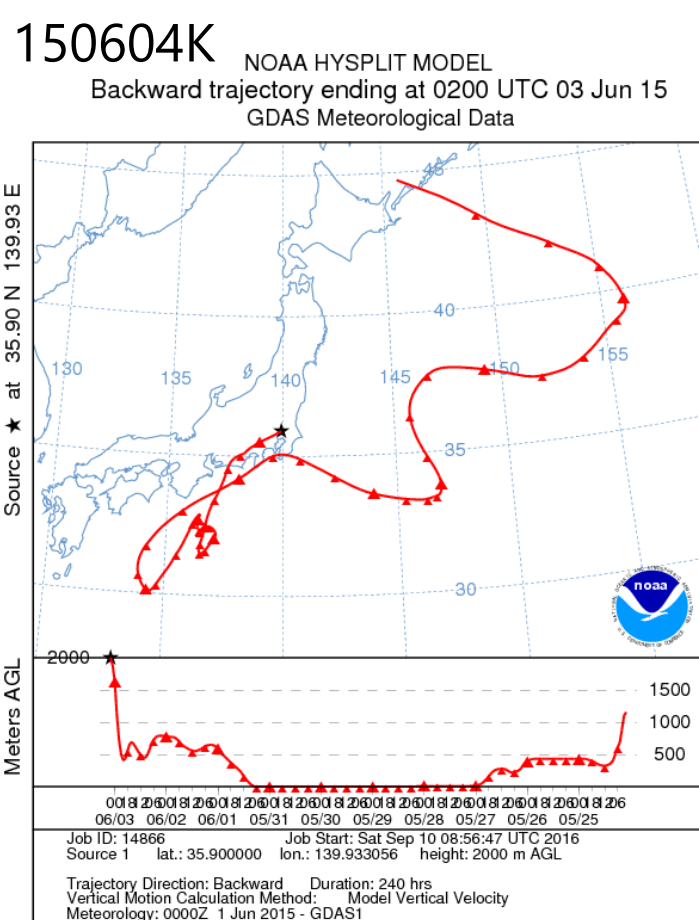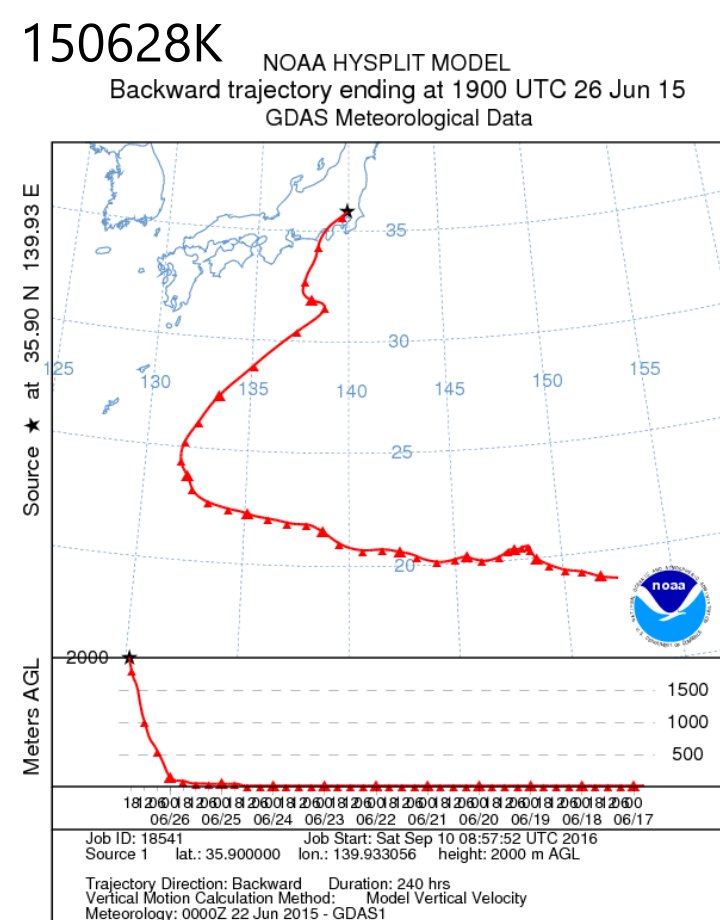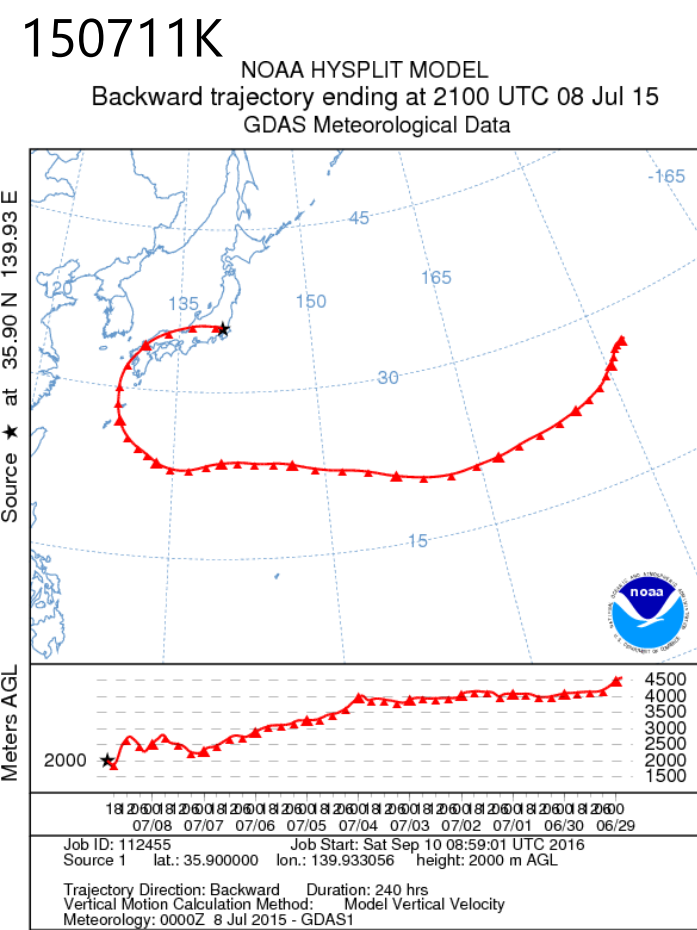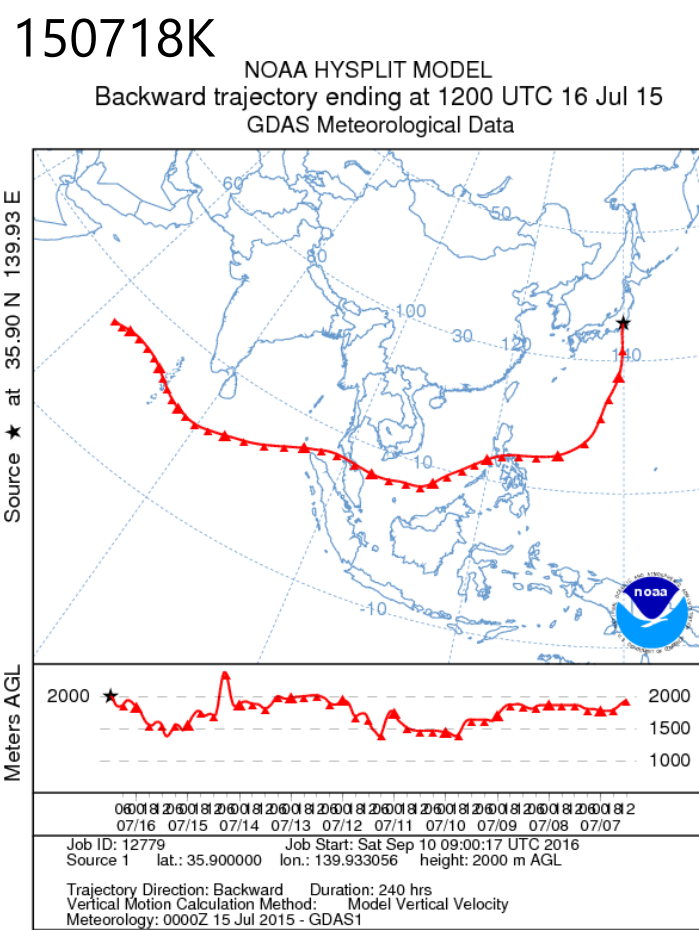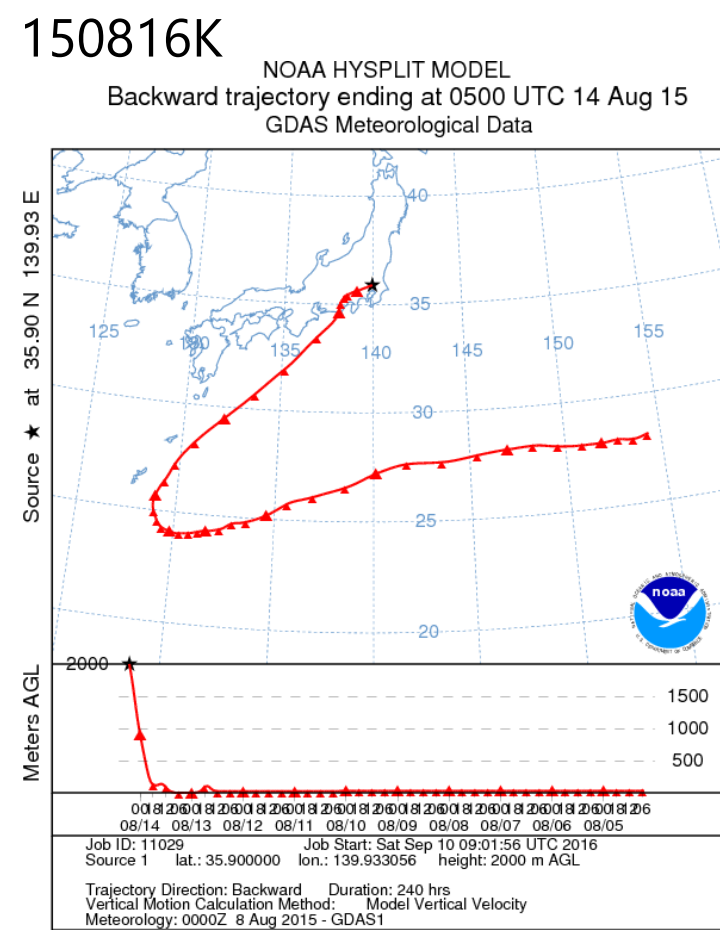

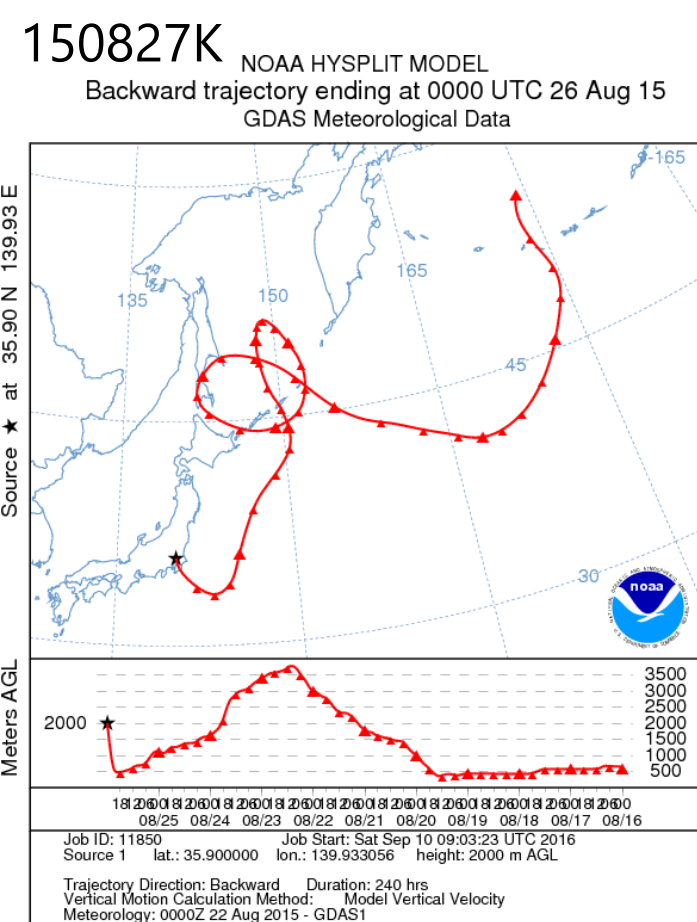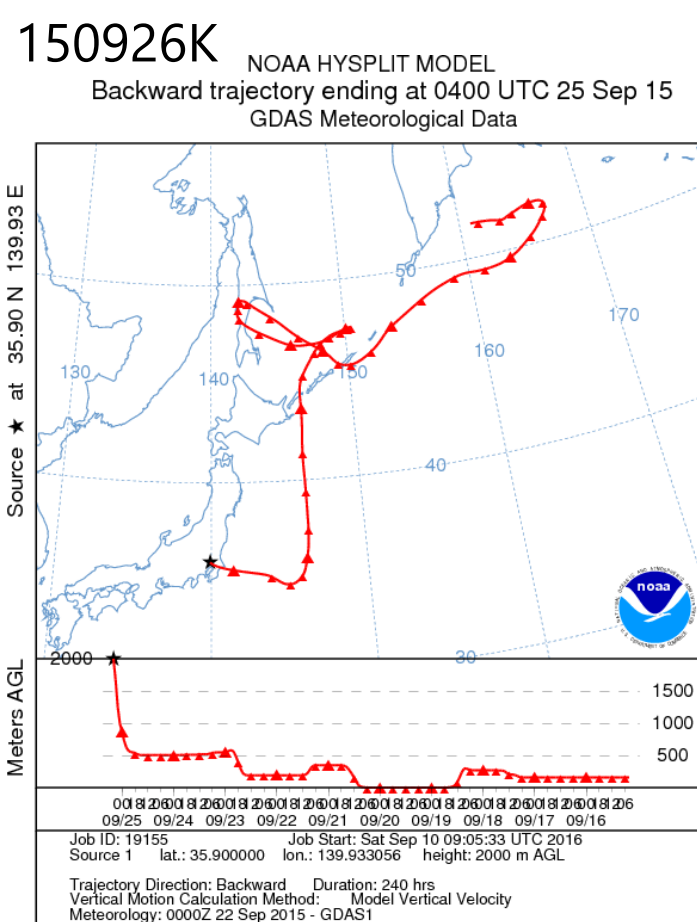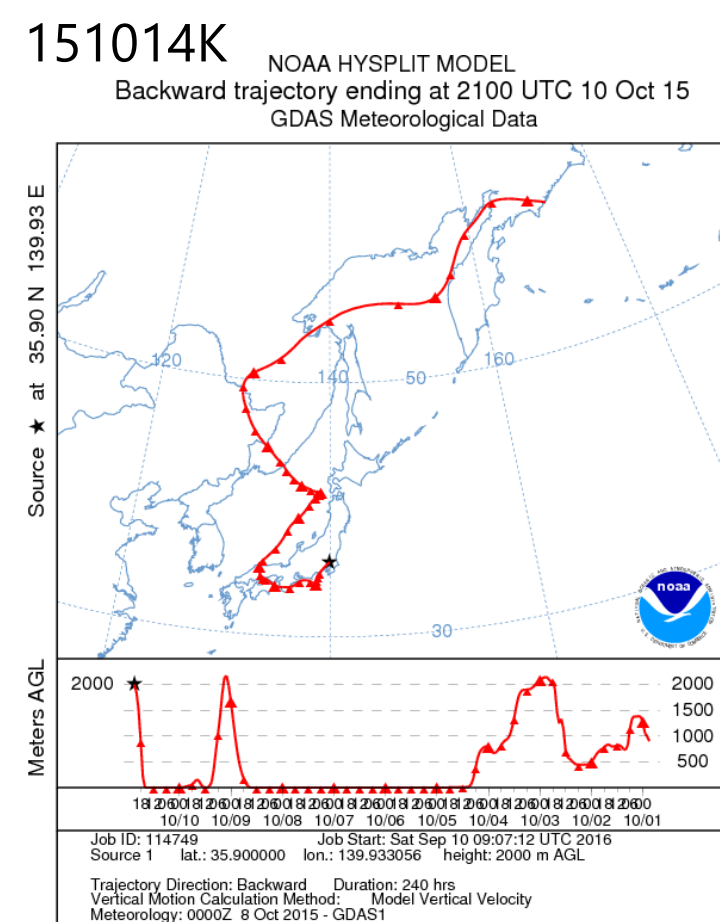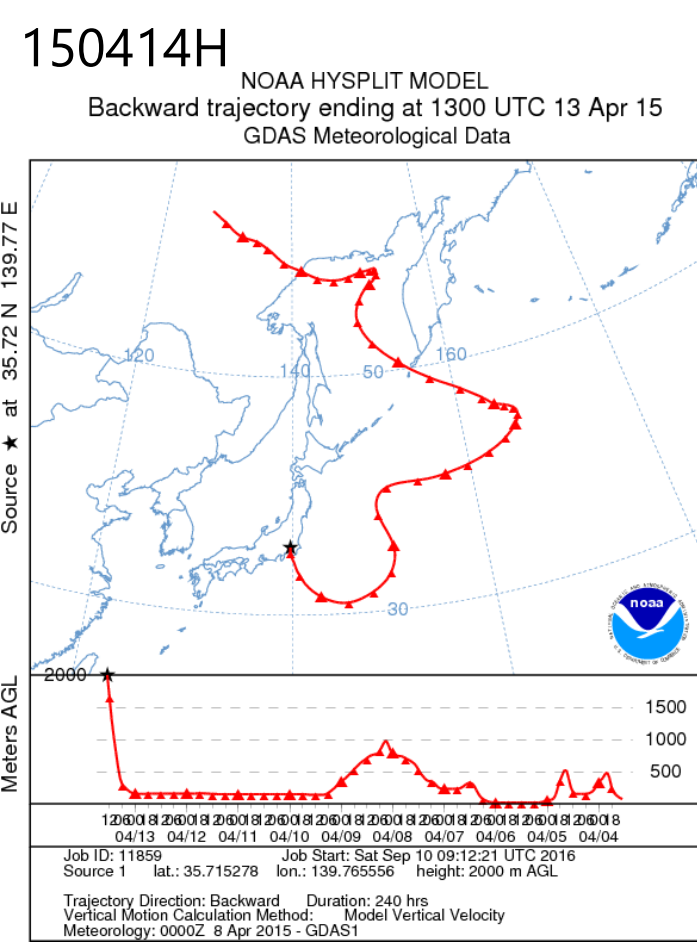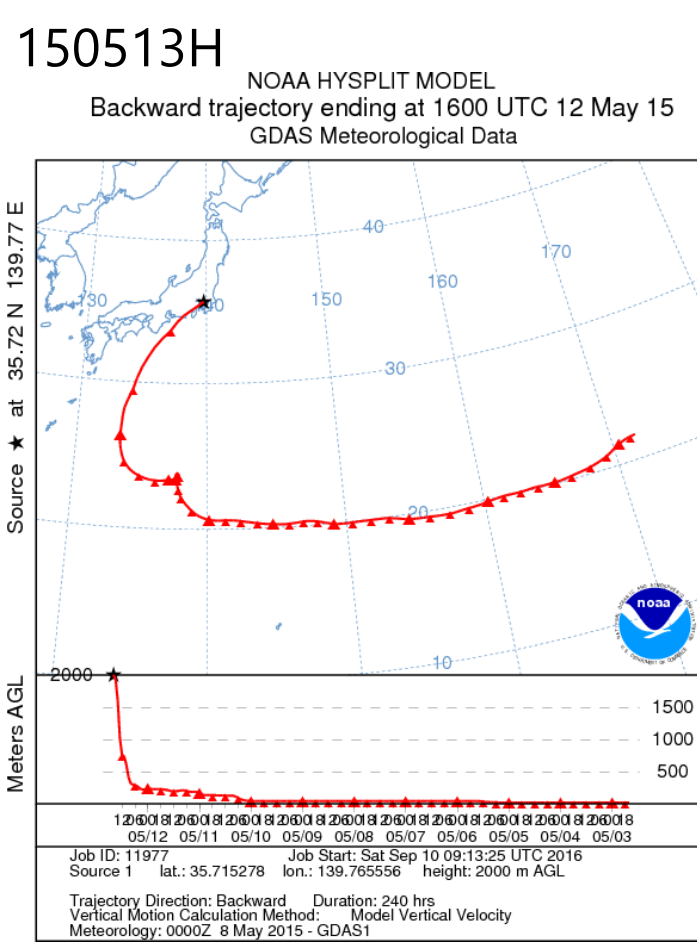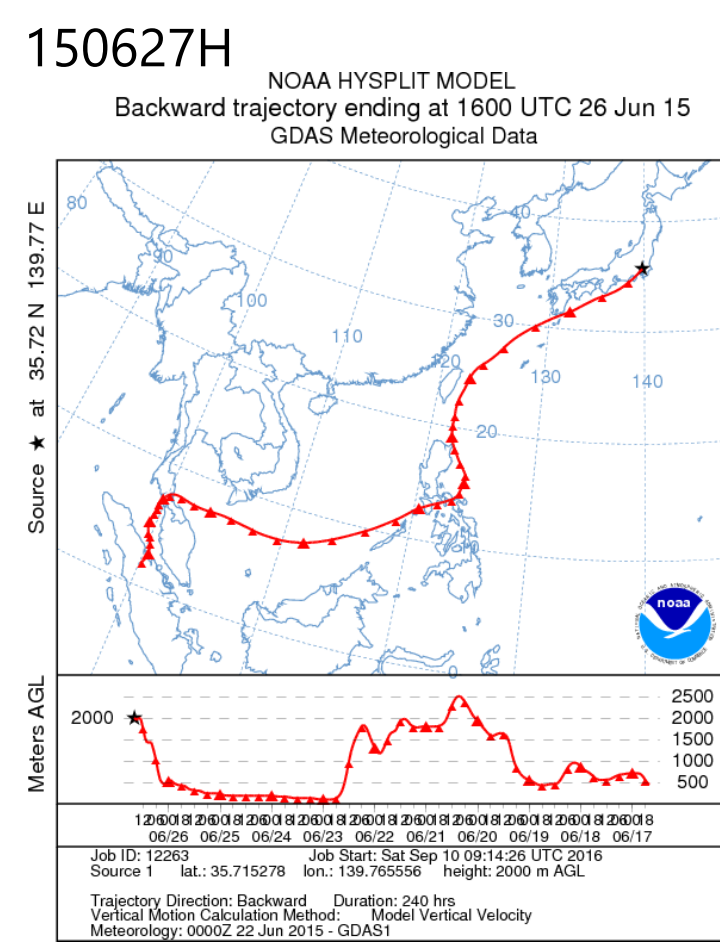

Supplement: Supplementary Figure S3 — The estimated air mass backward trajectories 240 h prior to precipitation events. [file Image3.PDF]

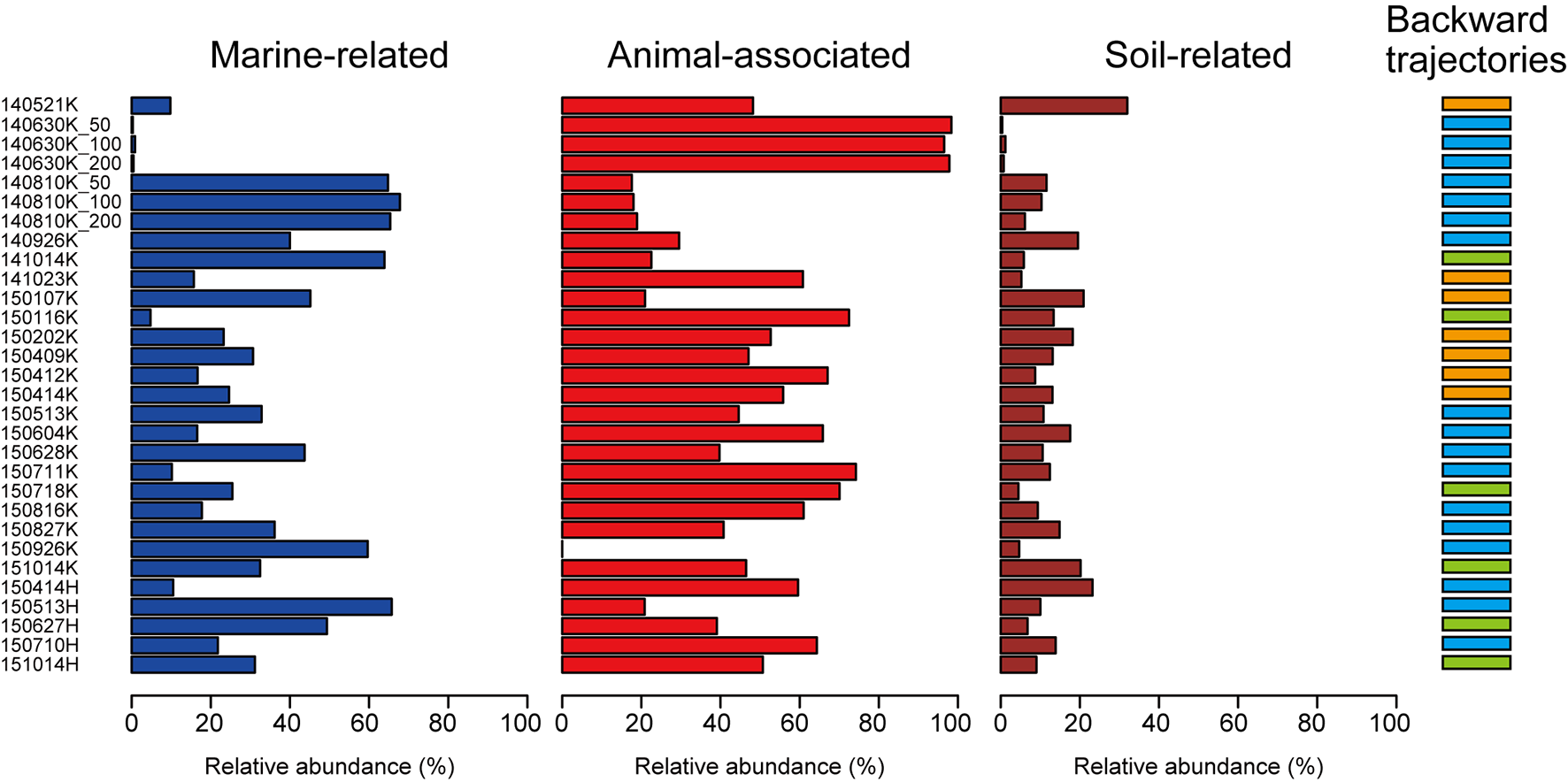

Supplement: Supplementary Figure S4 — Estimated ordinary habitats of precipitation microbes for three ecosystem groups. The abundance values in each ecosystem group are summation for habitats described below. Marine-related: “aquatic”, “marine”, “marine sediment”, “fish”, and “hot spring”; Animal-associated: “human”, “human gut”, “human lung”, “human nasal pharyngeal”, “bovine gut”, and “mouse gut”; and Soil-related: “hydrocarbon”, “rhizosphere”, “soil”, and “terrestrial.” The estimated route of the air mass before each precipitation event is indicated in the right column. [file Image4.TIF]

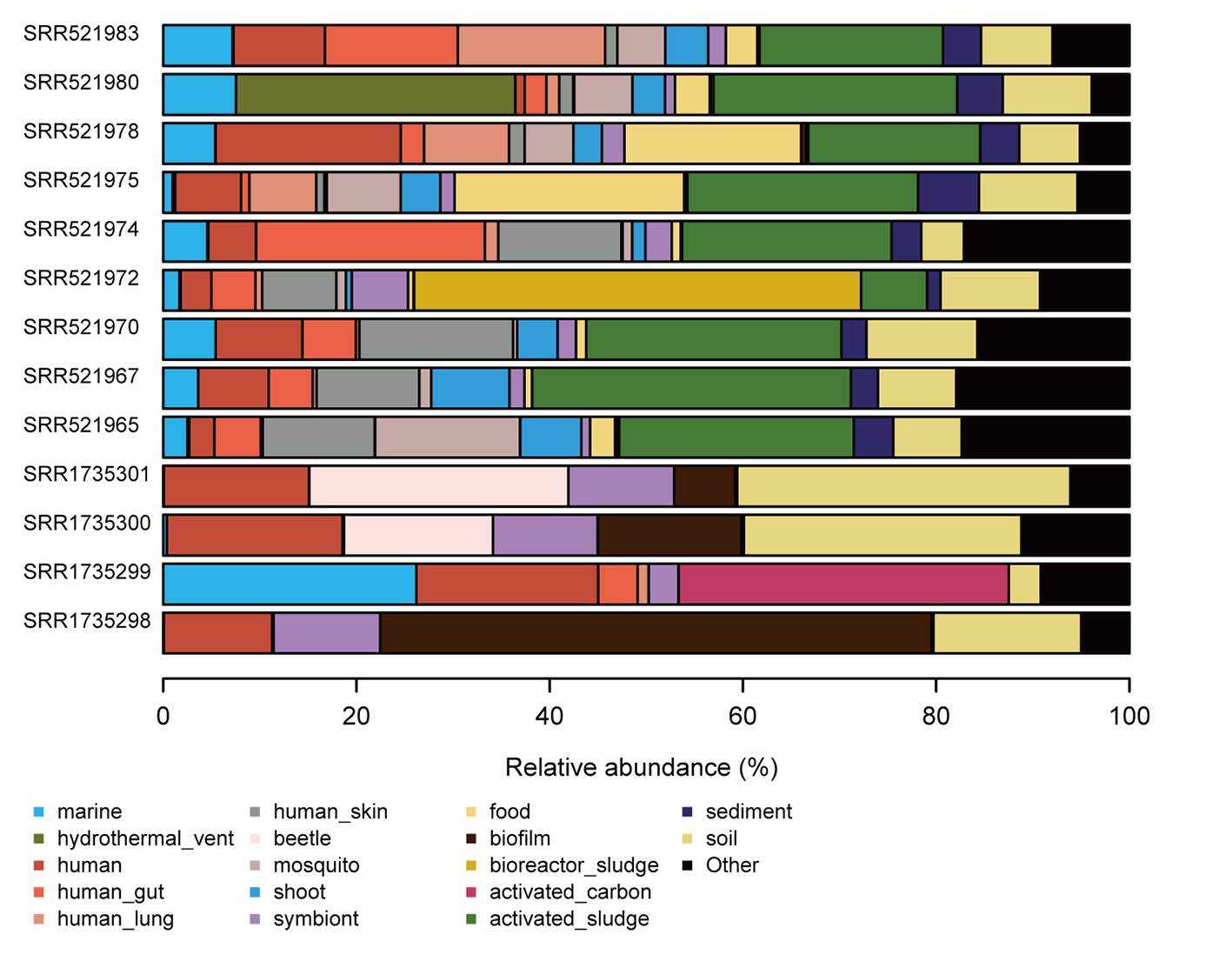

Supplement: Supplementary Figure S5 — Estimated ordinary habitats of microbes in aerosol and cloud water samples. Estimated ordinary habitats demonstrating <5% abundance were summarized as “Others.” [file Image5.TIF]
